# Structure-Activity Relationships of Synthetic Coumarins as HIV-1 Inhibitors

**DOI:** 10.1155/BCA/2006/68274

**Published:** 2006-02-23

**Authors:** I. Kostova, S. Raleva, P. Genova, R. Argirova

**Affiliations:** ^1^Department of Chemistry, Faculty of Pharmacy, Medical University, 2 Dunav Street, 1000 Sofia, Bulgaria; ^2^Department of Virology, National Center of Infectious and Parasitic Diseases, 44A Stoletor Street, 1233 Sofia, Bulgaria

## Abstract

HIV/AIDS pandemics is a serious threat to health and development of mankind, and searching for effective anti-HIV agents remains actual. Considerable progress has been made in recent years in the field of drug development against HIV. A lot of structurally different coumarins were found to display potent anti-HIV activity. The current review demonstrates the variety of synthetic coumarins having unique mechanism of action referring to the different stages of HIV replication. Recent studies based on the account of various synthetic coumarins seem to indicate that some of them serve as potent non-nucleoside RT-inhibitors, another as inhibitors of HIV-integrase or HIV-protease. The merits of selecting potential anti-HIV agents to be used in rational combination drugs design and structure-activity relationships are discussed.The scientific community is looking actively for new drugs and combinations for treatment of HIV infection effective for first-line treatment, as well as against resistant mutants. The investigation on chemical anti-HIV agents gives hope and optimism about it. This review article describes recent progress in the discovery, structure modification, and structure-activity relationship studies of potent anti-HIV coumarin derivatives.

## INTRODUCTION

AIDS remains an enormous health threat, although chemo-therapeutic agents have increased in number and effectiveness. Both nucleoside (AZT, DDI, DDC, D4T, 3TC) and non-nucleoside (nevirapine, delavirdine) HIV reverse transcriptase (RT) inhibitors and HIV protease (saquinavir, indinavir, ritonavir, nelfinavir) inhibitors have been licensed by the US FDA. Also, combination therapy of inhibitors of both groups results in undetectable levels of HIV in the blood of infected patients. However, despite this success, toxicity and, especially, drug resistance still present severe problems.

The replicative cycle of HIV is comprised of ten steps that may be adequate targets for chemotherapeutical intervention. Most of the substances identified as anti-HIV agents interfere with one of these steps of HIV replicative cycle. These steps are: (1) viral adsorption to the cell membrane, (2) fusion between the viral envelope and the cell membrane, (3) uncoating of the viral nucleocapsid, (4) reverse transcription of the viral RNA to proviral DNA, (5) integration of the proviral DNA into the cellular genome, (6) DNA replication, (7) transcription of the proviral DNA to RNA, (8) translation of the viral precursor mRNA to mature mRNA, (9) maturation of the viral precursor proteins by proteolysis, myristoylation, and glycosylation, and (10) budding, virion assembly, and release. Step 4, a key step in the replicative cycle of retroviruses, which makes it distinct from the replicative cycle of other viruses, is the reverse transcription catalyzed by reverse transcriptase. Another target for therapeutic intervention is step 9, particularly the proteolysis of precursor proteins by HIV protease. The majority of chemotherapeutic strategies have, therefore, focused on the development of retroviral enzyme inhibitors.

The US Food and Drug Administration (FDA) has approved a number of anti-HIV drugs for clinical use. However, these medications have limitations such as high cost, decreased sensitivity due to the rapid emergence of drug-resistant mutants, and adverse effects like peripheral neuropathy, bone marrow suppression, and anemia [1]. Thus, more effective and less toxic anti-HIV agents are still needed. In addition, alternative approaches, including herbal therapies after long-term screening of plant extracts, particularly anti-infective or immunomodulating medicinal herbs and the structural modification of lead compounds, have been attempted.

Coumarins, an old class of compounds, are naturally occurring benzopyrene derivatives. A lot of coumarins have been identified from natural sources, especially green plants. The pharmacological and biochemical properties and therapeutic applications of simple coumarins depend upon the pattern of substitution. Coumarins have attracted intense interest in recent years because of their diverse pharmacological properties.

Coumarins comprise a group of natural compounds found in a variety of plant sources. The very long association of plant coumarins with various animal species and other organisms throughout evolution may account for the extraordinary range of biochemical and pharmacological activities of these chemicals in mammalian and other biological systems. The coumarins that were studied have divers biological properties and various effects on the different cellular systems. A lot of biological parameters should be evaluated to increase our understanding of mechanisms by which these coumarins act. Coumarins have important effects in plant biochemistry and physiology, acting as antioxidants, enzyme inhibitors, and precursors of toxic substances. In addition, these compounds are involved in the actions of plant growth hormones and growth regulators, the control of respiration, photosynthesis, as well as defense against infection. The coumarins have long been recognized to possess anti-inflammatory, antioxidant, antiallergic, hepatoprotective, antithrombotic, antiviral, and anticarcinogenic activities. The hydroxycoumarins are typical phenolic compounds and, therefore, act as potent metal chelators and free radical scavengers. They are powerful chain-breaking antioxidants. The coumarins display a remarkable array of biochemical and pharmacological actions, some of which suggest that certain members of this group of compounds may significantly affect the function of various mammalian cellular systems.

The coumarins are extremely variable in structure, due to the various types of substitutions in their basic structure, which can influence their biological activity. A careful structure-system-activity-relationship study of coumarins should be conducted.

### Reverse transcriptase inhibitors

**Scheme 1:**
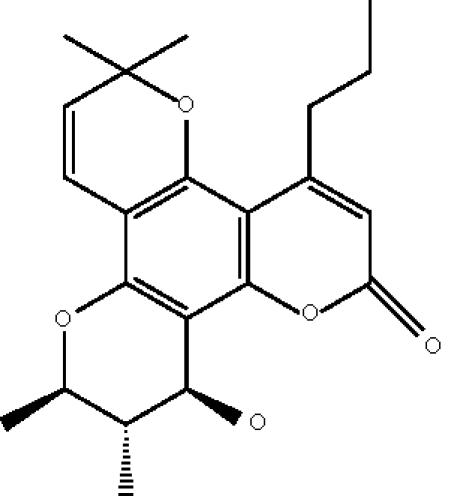
(+)-calanolide A.

*Synthetic calanolides*

(+)-calanolide A (Scheme 1), (+)-[10R, 11S, 12S]-10,11-trans-dihydro-12-hydroxy-6,6,10,11-tetramethyl-4-propyl-2H,6H-benzo[1,2-b:3,4-b′:5,6-b″]tripyran-2-one, is a novel non-nucleoside reverse transcriptase inhibitor (NNRTI) with potent activity against HIV-1 [2–4]. The compound was first isolated from a tropical tree (*Calophyllum lanigerum*) in Malaysia [4]. Due to low availability of naturally occurring (+)-calanolide A, a total synthesis of this polycyclic coumarin was developed to provide material for preclinical and clinical research [4, 5]. Structural biology studies and enzyme kinetic experiments bear out the unique anti-HIV properties of calanolide A. In particular, calanolide A is active against viral isolates with the Y181C amino acid mutation in the reverse transcriptase of HIV-1. This is a commonly observed mutation identified in both laboratory and clinical viral isolates and is associated with high-level resistance to most other NNRTIs. However, viral isolates that contain multiple AZT-resistant mutations and the Y181C mutation are actually hypersensitive to the antiviral activity of calanolide A. When tested in vitro in combination with a range of nucleoside analogues, protease inhibitors and NNRTIs, calanolide A demonstrates additive to synergistic anti-HIV activity.

**Scheme 2:**
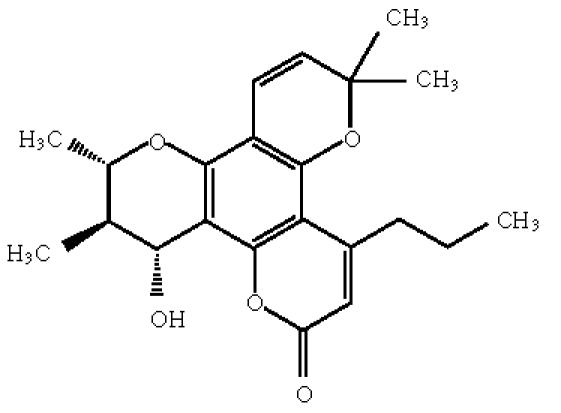
(−)-calanolide A.

The anti-HIV agent (+/−)-calanolide A has been synthesized [5–7] in a five-step approach starting with phloroglucinol, which includes Pechmann reaction, Friedel-Crafts acylation, chromenylation with 4,4-dimethoxy-2-methylbutan-2-ol, cyclization, and Luche reduction. Cyclization of chromene to chromanon was achieved by employing either acetaldehyde diethyl acetal or paraldehyde in the presence of trifluoroacetic acid and pyridine or PPTS. Luche reduction of chromanone at lower temperature preferably yielded (+/−)-calanolide A. The synthetic (+/−)-calanolide A has been chromatographically resolved into its optically active forms, (+)- and (−)-calanolide A (Scheme 2). The anti-HIV activities for synthetic (+/−)-calanolide A, as well as resultant (+)- and (−)-calanolide A, have been determined. Only (+)-calanolide A accounted for anti-HIV activity, which was similar to the data reported for the natural product, and (−)-calanolide A was inactive.

In order to examine the structure-activity relationships of the trans-10,11-dimethyldihydropyran-12-ol ring (designated ring C), a series of structural analogues were prepared and evaluated using cell cytopathicity assay (XTT) [14]. Removal of the 10-methyl group resulted in decreased activity, with only one epimer exhibiting anti-HIV activity. Substituting the 10-methyl group with an ethyl chain maintained anti-HIV activity, with only a 4-fold reduction in potency relative to racemic calanolide A. Substitution of the 10-methyl group with an isopropyl moiety completely eliminated the anti-HIV activity. Addition of an extra methyl group at either the 10- or 11-position maintained the basic stereochemical features of the parent calanolide system while removing the chirality at the respective carbon, but resulted in decreased activity relative to calanolide A. In the above examples, analogues containing a *cis* relationship between the 10- and 11-alkyl moieties were completely devoid of activity. Synthetic intermediates in which the 12-hydroxyl group was in the ketone oxidation state exhibited suppressing anti-HIV activity, with EC_50_ values only 5-fold less potent than that of calanolide A for both the 10,11-*cis* and -*trans* series. These ketones represent the first derivatives in the calanolide series to exhibit anti-HIV activity while not containing a 12-hydroxyl group. Analogues which showed anti-HIV activity in the CEM-SS cytoprotection assay were further confirmed to be inhibitors of HIV-1 reverse transcriptase. The synthesis of calanolide A has been reported in [15–17].

**Scheme 3:**
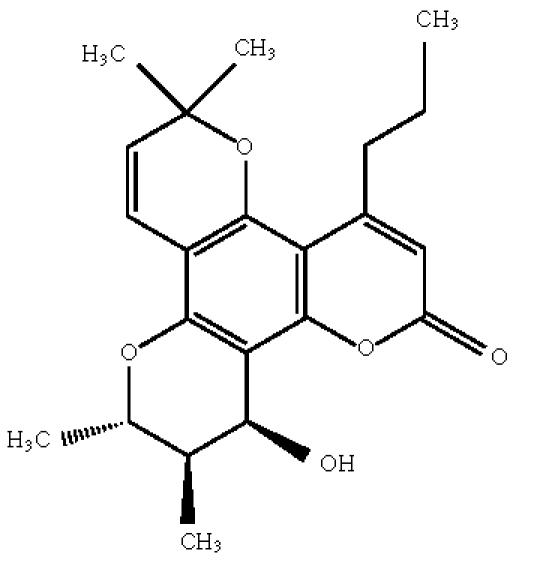
(−)-calanolide B.

The delta 7,8 olefinic linkages within (+)-calanolide A and (−)-calanolide B (Scheme 3) were catalytically reduced to determine impact on the anti-HIV activity of the parent compounds [18]. In addition, a series of structure modifications of the C-12 hydroxyl group in (−)-calanolide B was made to investigate the importance of that substituent to the HIV-1 inhibitory activity of these coumarins. A total of 14 analogues were isolated or prepared and compared to (+)-calanolide A and (−)-calanolide B in the National Cancer Institute. While none of the compounds showed activity superior to the unmodified leads, some structure-activity requirements were apparent from the relative anti-HIV potencies of the various analogues [19–22]. The synthesis of the isomers of calanolide A has been reported in [23, 24].

NMR spectra of synthetic structures corresponding to those initially reported for natural compounds calanolide C and calanolide D showed some subtle differences from those of the natural products. Further analysis has resulted in revision of the structures of the natural compounds, now renamed as pseudocalanolides C and D. The absolute stereochemistry of pseudocalanolide C was established as [6S, 7S, 8R] using the modified Mosher's method [25].

**Scheme 4:**
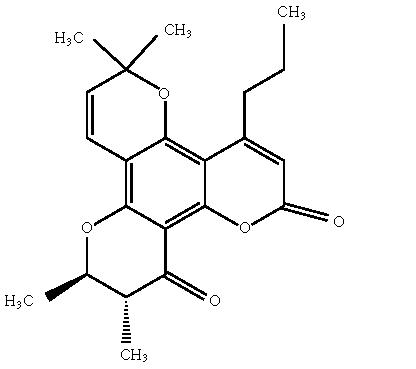
(+)-12-oxocalanolide.

The three chromanone derivatives (+)-, (−)-, and (+/−)-12-oxocalanolide A (Scheme 4) were evaluated for in vitro antiviral activities against HIV and simian immunodeficiency virus (SIV). The compounds were determined to be inhibitors of HIV-1 reverse transcriptase (RT) and exhibited activity against a variety of viruses selected for resistance to other HIV-1 non-nucleoside RT inhibitors. They are the first reported calanolide analogues capable of inhibiting SIV [26].

*Synthetic DCK analogues*

Numerous plant-derived compounds have been evaluated for inhibitory effects against HIV replication, and some coumarins have been found to inhibit different stages in the HIV replication cycle. The review article [27] describes recent progress in the discovery, structure modification, and structure-activity relationship studies of potent anti-HIV coumarin derivatives. A dicamphanoyl-khellactone (DCK) analogue, which was discovered and developed in author's laboratory, and calanolide A are currently in preclinical studies and clinical trials, respectively.

**Scheme 5:**
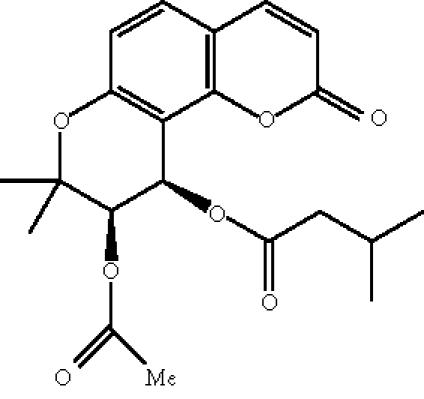
Suksdorfin.

**Scheme 6:**
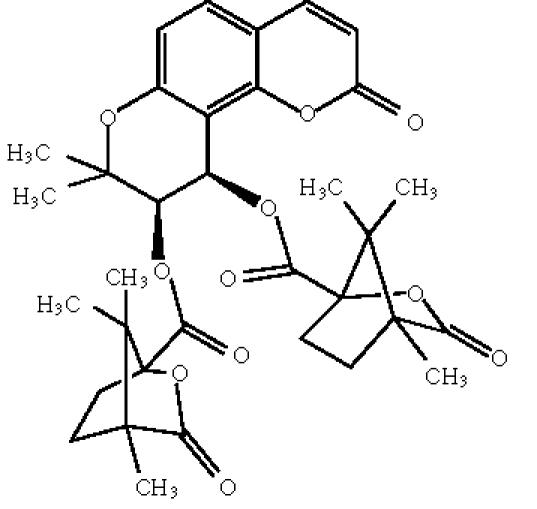
Dicamphanoyl-khellactone.

Through a bioactivity-directed search for plant-derived, naturally occurring compounds, the lead compound sukudorfin was isolated from the fruit of Lomatium suksdorfii and its structure was identified. Sukudorfin (Scheme 5) inhibited HIV-1 replication in H9 lymphocytes (the EC_50_ and the therapeutic index—TIs for some coumarins are reviewed in Table 1). The discovery of suksdorfin led to the synthetic compounds and the most promising lead compound was 3,4-di-O-(s)-(−)-camphanoyl-(3′R, 4′R)-(+)-*cis*-khellactone (or DCK) Scheme 6, which showed extremely potent activity [1].

**Scheme 7:**
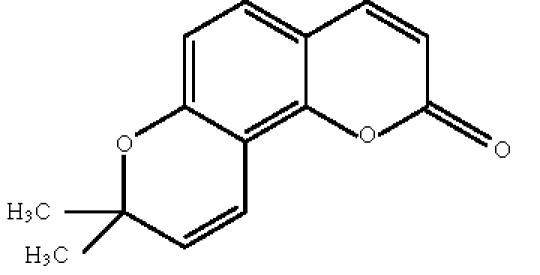
Seselin.

Forty two dihydroseselins based on the structure of suksdorfin were synthesized in order to evaluate their anti-HIV activity [8]. These synthetic derivatives include 3′,4′-di-O-acyl- and 3′- or 4′-O-acyl-*cis*-dihydroseselins and 3′,4′-trans-dihydroseselins with O-acyl and/or O-alkyl groups at the 3′ and 4′ positions. Two 4′-azido and three 4′-alkylamido derivatives were also prepared. By using optically pure reagents, three pairs of diastereoisomers were synthesized and separated as optically pure compounds. Together with the above synthetic derivatives, seselin Scheme 7 and (+/−)-*cis*-, (+)-*cis*-, and (+/−)-*trans*-dihydroseselin-3′,4′-diol were also tested for anti-HIV activity in vitro. An optically pure compound, 3′,4′-di-O-(−)-camphanoyl-(+)-*cis*-khellactone, showed potent inhibitory activity and remarkable selectivity against HIV replication. The EC_50_ value and in vitro therapeutic index (TI) of 3′,4′-di-O-(−)-camphanoyl-(+)-*cis*-khellactone are better than those shown by AZT (Table 1). In addition, compound 3′,4′-di-O-(−)-camphanoyl-(+)-*cis*-khellactone is also active against HIV replication in a monocytic cell line and in peripheral blood mononuclear cells (PBMCs). In vitro assay indicated that, like compound suksdorfin, compound 3′,4′-di-O-(−)-camphanoyl-(+)-*cis*-khellactone is not an inhibitor of HIV-1 reverse transcriptase. Moreover, the anti-HIV activity of 3′,4′-di-O-(−)-camphanoyl-(+)-*cis*-khellactone is stereoselective as its three diastereoisomers are at least 10,000 times less active. Since other synthetic dihydroseselin derivatives with different substituents or without any substituents are inactive or are active only at much higher concentration, the antiviral potency of 3′,4′-di-O-(−)-camphanoyl-(+)-*cis*-khellactone could be associated with the camphanoyl moieties of its structure. Therefore, compound 3′,4′-di-O-(−)-camphanoyl-(+)-*cis*-khellactone represents a unique coumarin structure with promising anti-HIV activity.

DCK lactam analogues were synthesized [9] and evaluated in H9 cells. 4-methyl-DCK lactam exhibited potent anti-HIV activity (Table 1).

A series of disubstituted 3′,4′-di-O-(S)-camphanoyl-(+)-*cis*-khellactone (DCK) analogues were synthesized [10] and evaluated for inhibition of HIV-1 replication in H9 lymphocytes. 5-methoxy-4-methyl DCK was the most promising compound. As seen in Table 1, its parameters were much better than those of lead compound DCK. Another six disubstituted DCK analogues were more potent than AZT but less active than DCK. Conformational analysis suggested that resonance of the coumarin system is an essential structural feature for potent anti-HIV activity. Steric compression of C(4) and C(5) substituents of the coumarin moiety can reduce the overall planarity and thus resonance of the coumarin nucleus, resulting in a decrease or lack of anti-HIV activity.

To explore the structural requirements of (+)-*cis*-khellactone derivatives as novel anti-HIV agents, 24 monosubstituted 3′,4′-di-O-(S)-camphanoyl-(+)-*cis*-khellactone (DCK) derivatives were synthesized in enantiometrically pure form [12]. These compounds included four isomeric monomethoxy analogues, four isomeric monomethyl analogues, four 4-alkyl/aryl-substituted analogues, and twelve 4-methyl-(+)-*cis*-khellactone derivatives with varying 3′,4′-substituents. These (+)-*cis*-khellactone derivatives were screened in acutely infected H9 lymphocytes. The results demonstrated that the (3′R, 4′R)-(+)-*cis*-khellactone skeleton, two (S)-(−)-camphanoyl groups at the 3′- and 4′-positions, and a methyl group on the coumarin ring, except at the 6-position, were optimal structural moieties for anti-HIV activity. EC_50_ and TI values for 3-methyl-, 4-methyl-, and 5-methyl-3′,4′-di-O-(S)-camphanoyl-(3′R, 4′R)-(+)-*cis*-khellactone are shown in Table 1. Furthermore, 4-methyl-, and 5-methyl-3′,4′-di-O-(S)-camphanoyl-(3′R, 4′R)-(+)-*cis*-khellactone also showed potent inhibitory activity in CEM-SS cell line, and most monosubstituted DCK analogues were less toxic than DCK.

Six 3-substituted 3′,4′-di-O-(S)-camphanoyl-(+)-*cis*-khellactone derivatives were synthesized from 3-methyl DCK. 3-hydroxymethyl DCK exhibited potent anti-HIV activity in H9 lymphocytes shown in Table 1. These values are similar to those of DCK and better than those of AZT [13].

In additional structure-activity-relationship (SAR) studies [1], the carbonyl oxygen of DCK was replaced with a sulfur atom. This bioisostere was less potent but also less cytotoxic than DCK. The 4-methyl, -propyl, and -benzyl analogues were also prepared; the order of activity was methyl > H > propyl > benzyl. 4-methyl-3′,4′-di-O-(−)-camphonyl-(+)-*cis*-khelthiolactone exhibited extremely potent anti-HIV activity (Table 1).

To enhance the water solubility and oral bioavailability of DCK analogues, 12 new mono- and disubstituted (3′R, 4′R)-3′,4′-di-O-(S)-camphanoyl-(+)-*cis*-khellactone (DCK) analogues were synthesized and evaluated for inhibition of HIV-1 replication in H9 lymphocytes [11]. 3-hydroxymethyl-4-methyl-DCK exhibited significant anti-HIV activity in H9 lymphocytes and even less in primary peripheral blood mononuclear cells (Table 1). Although this compound was not as potent as 4-methyl-DCK and 3-bromomethyl-4-methyl-DCK, it provides increased water solubility and possible linkage to other moieties. Of particular note, 3-hydroxymethyl-4-methyl-DCK exhibits moderate oral bioavailability (15%) when administered as a carboxymethylcellulose suspension to rats, whereas 4-methyl-DCK is not orally bioavailable in the same formulation. Further studies on mechanism of action suggest that 3-hydroxymethyl-4-methyl-DCK inhibits the production of double-stranded viral DNA from the single-stranded DNA intermediate. In addition, 3-bromomethyl-4-methyl-DCK is the most potent compound in this series of new analogues with EC_50_ and TI values shown in Table 1. Thus, further modification at the 3-position of the coumarin ring can improve the potency of new DCK analogues.

### Other reverse transcriptase inhibitors

Ten different pyranone-related substituents (chromones or coumarins) were covalently linked to the 5′ end of various oligonucleotides (ODNs). The interaction of these compounds with HIV-1 RT was analyzed. A different behavior was found to depend on the structure of the oligonucleotide derivatives. Some compounds activated the enzyme at relatively low concentrations (0.1–0.5 *μ*M), followed by inhibition of the activity at higher concentrations (5–20 *μ*M), whereas others behave just as inhibitors. Because the presence of some coumarin or chromone derivatives conjugated to ODNs enhanced the interaction with RT, Martyanov et al [28] analyzed the capacity of such ODN derivatives to be used as primers. The introduction of a chromone derivative, the 2-[3-(aminopropyl)amino]-8-isopropyl-5- methyl-4-oxo-4H-1-benzopyran-3-carbaldehyde], and a coumarin derivative, the 1-(3-aminopropoxy)-2-ethyl-3H-naphto [2,1-b] pyran-3-one, into the 5′ end of a noncomplementary ODN allowed these compounds to be used as primers. In the case of complementary primers, the presence of conjugated derivatives enhanced the affinity with Km values that were two to three orders of magnitude lower than that of a complementary primer of the same length. After addition of a ddT-unit to the 3′-terminal end of the ODN, some of these primers became very effective inhibitors of RT with Ki values in the nanomolar range. Martyanov et al [29] have carried out a comparison of Km and Vmax values for various primers in the polymerization reaction catalyzed by the HIV-1 RT. The affinity of RT for complementary d(pT)6 containing two different 5′-end pyranone derivatives was two to three orders of magnitude higher (Km = 3–15 nM) than that of d(pT)6 (Km = 12.6 mM). ODNs noncomplementary to poly(A) template were not elongated by RT. However, derivatives of d(CAGGTG) containing the 5′-terminal chromone and coumarin related groups were efficient primers showing Km (30–300 nM) and Vmax (75%–93%) values comparable with that for d(pT)10 (800 nM; 100%). The [d(CAGGTG)]ddT ODN derivatives were effective inhibitors of RT. The primer function of derivatives of noncomplementary ODNs appears to be due to the additional interactions of their 5′-terminal groups with the enzyme tRNA-binding site.

Toddacoumaquinone is a coumarin-naphthoquinone dimer, and it was synthesized through Diels-Alder reaction between 8-(1-acetoxy-3-methyl-1, 3-butadienyl)-5, 7-dime-thoxycoumarin and 2-methoxy-1, 4-benzoquinone [30]. The activities of Toddacoumaquinone against several viruses were examined. A weak activity (EC_50_ = 10 *μ*gr/mL) was observed against *Herpes simplex* virus type 1 and 2 (HSV-1 and HSV-2), but no activity was seen against HIV-1.

A new series of 3,5-bis(arylidene)-4-piperidones, as chal- cone analogues carrying variety of aryl and heteroaryl groups, pyrazolo[4,3-c]pyridines, pyridolo[4,3-c]pyrimi-dines, and pyrido[4,3-c]pyridines, carrying an arylidene moiety, and a series of pyrano[3,2-c]pyridines, as flavone and coumarin isosteres, were synthesized [31] and screened for their in vitro antiviral and antitumor activities at the National Cancer Institute. Several compounds proved to be active against HSV-1, while other compounds showed moderate activity against HIV-1. The pyrano[3,2-c]pyridines heterocyclic system proved to be the most active antitumors among the investigated heterocycles.

**Scheme 8:**
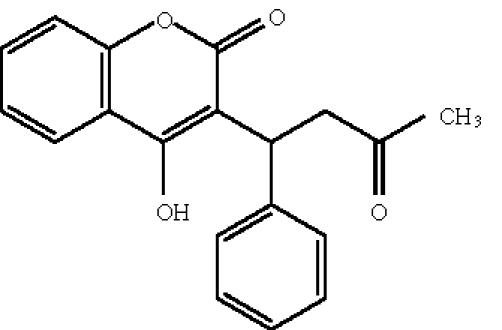
Warfarin.

**Scheme 9:**
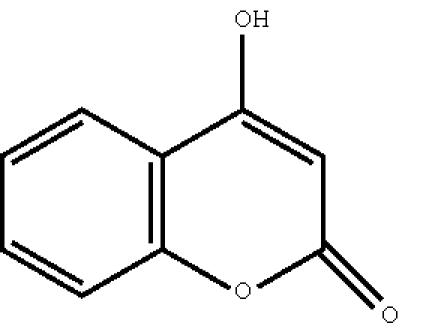
4-hydroxycoumarin.

**scheme 10:**
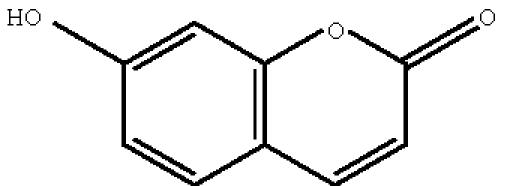
Umbelliferone.

A single dose of coumarin derivatives, warfarin (Scheme 8), 4-hydroxycoumarin (Scheme 9), and umbelliferone (Scheme 10), added at the time of inoculation either by free virus or by contact with U1 monocytes exhibited a dose-dependent inhibitory effect on viral replication in target MOLT-4 lymphocytes observable even at 5 days after infection [32]. In addition, marked decrease of HIV-1 p24 release and reduction in RT activity was observed when chronically HIV-infected ACH-2 lymphocytes were treated with coumarins (ED_50_ range 10^−6^−10^−9^ mol/L). However, the intracellular composition of HIV-1 core proteins in drug-exposed cells was not modified. Results suggest that although no complete inhibition of viral production has been observed in vitro, this class of drugs may present potential interest as antiviral agents.

### Integrase inhibitors

The structures of a large number of HIV-1 integrase inhibitors have in common two aryl units separated by a central linker. Frequently, at least one of these aryl moieties must contain 1,2-dihydroxy substituents in order to exhibit high inhibitory potency. The ability of o-dihydroxy-containing species to undergo in situ oxidation to reactive quinones presents a potential limitation to the utility of such compounds. The report of tetrameric 4-hydroxycoumarin-derived inhibitor provided a lead example of an inhibitor which does not contain the catechol moiety. Tetrameric 4-hydroxycoumarin-derived inhibitor represents a large, highly complex, yet symmetrical molecule. It was the purpose of the study [33] to determine the critical components of tetrameric 4-hydroxycoumarin-derived inhibitor, and if possible to simplify its structure while maintaining potency. In the study, dissection of tetrameric 4-hydroxycoumarin-derived inhibitor (IC_50_ = 1.5 *μ*M) into its constituent parts showed that the minimum active pharmacophore consisted of a coumarin dimer containing an aryl substituent on the central linker methylene. However, in the simplest case in which the central linker aryl unit consisted of a phenyl ring (IC_50_ = 43 *μ*M), a significant reduction in potency resulted by removing two of the original four coumarin units. Replacement of this central phenyl ring by more extended aromatic systems having higher lipophilicity improved potency, as did the addition of 7-hydroxy substituents to the coumarin rings. Combining these latter two modifications resulted in compounds such as 3,3′-(2-naphthalenomethylene)bis[4,7-dihydroxycoumarin] (IC_50_ = 4.2 *μ*M) which exhibited nearly the full potency of the parent tetramer, yet were structurally much simpler.

The most important method that can be applied to develop new anti-AIDS compounds is computer-assisted drug design (CADD). The used method involves traditional or classic QSAR and 3D QSAR. In the traditional approach to QSAR, the chemical structure can be described with experimental and theoretical steric, electronic, and hydrophobic parameters. 3D QSAR methods were developed as an alternative to traditional QSAR to describe molecules. The results indicated that the flavonoids with hydroxyl groups at C-5 and C-7 in the A-ring, and with a C-2-C-3 double bond were the most potent HIV growth inhibitors [33]. According to the above information on structure-activity relationships, structure modification methods can be also used for flavonoid lead compounds, which are derived from plants with possible anti-HIV activity. As a potential target, the heteroatom in position 1 of the C-ring of the flavonoid compounds has been considered. Therefore, similar or even new biological activities could be anticipated when the oxygen of bioactive flavonoids is replaced by another atom such as nitrogen or sulfur, which lines up closely with oxygen in the periodic table. Thus, a series of 5,6,7,8-subsituted-2-phenylthio-chromen-4-ones has been synthesized and evaluated for anti-HIV activity [34]. Among them, one new compound was the most active (IC_50_ value of 0.65 *μ*M) against HIV in acutely infected H9 lymphocytes, and had a TI of approximately 5. A systematic series of chemically modified coumarin dimers has been synthesized and tested for their inhibitory activity against HIV-1 integrase. Mao et al [35] observed that modified coumarin dimers containing hydrophobic moiety on the linker display potent inhibitory activities.

### Protease inhibitors

**scheme 11:**
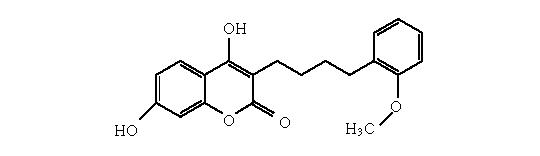
4,7-dihydroxy-3-[4-(2-methoxyphenyl)butyl]-2H-chromen-2-one.

HIV-1 protease has been identified as a significant target enzyme in AIDS research. While numerous peptide-derived inhibitors have been described, the identification of a nonpeptide inhibitor remains an important goal. Using an HIV-1 protease mass screening technique, 4-hydroxy-3-(3-phenoxypropyl)-2H-1-benzopyran-2-one was identified as a nonpeptide competitive inhibitor of the enzyme [36]. Employing a Monte Carlo-based docking procedure, the coumarin was docked in the active site of the enzyme, revealing a binding mode that was later confirmed by the X-ray crystal analysis. Several analogues were prepared to test the binding interactions and improve the overall binding affinity. The most active compound in the study was 4,7-dihydroxy-3-[4-(2-methoxyphenyl)butyl]-2H-1-benzo-pyran-2-one (Scheme 11).

The screening of the HIV-1 protease (PR) inhibitory activity (IC_50_) of various substituted 3-phenyl-4-hydroxy- coumarins, 3-benzyl-4-hydroxycoumarins, 3-phenoxy-4-hy-droxy-coumarins, 3-benzenesulfonyl-4-hydroxycoumarins, and 3-(7-coumarinyloxy)-4-hydroxycoumarins was performed [37]. The data indicate the importance of substituents at positions 5 and 7 of the coumarin ring on the inhibitory potency of the HIV-1-PR.

The interaction of novel series of synthetic inhibitors with various serine proteases (leukocyte elastase, thrombin, cathepsin G, chymotrypsin, plasminogen activators, and plasmin) and an aspartic protease (HIV-1 protease) were studied [38]. Various aspects were analyzed: mechanism of action, structure-activity relationships, and in some cases, molecular modeling, and biological evaluation. Functionalized cyclopeptides and N-aryl azetidin-2-ones behaved as suicide substrates acting specifically on trypsin-like proteases (thrombin or urokinase) and elastases, respectively. Novel hydrazinopeptides acted as reversible inhibitors of elastases. Coumarin derivatives inactivated very efficiently chymotrypsin-like proteases (k(inact)/K(I) = 760,000 M^−1^. s^−1^). Inhibitors of HIV-1 protease acting either as inactivators or dimerization inhibitors are under investigation. The inhibitors described above are useful for elucidating the biological roles of the target enzymes and constitute potential drugs.

## CONCLUSION

Coumarins comprise a vast array of biologically active compounds ubiquitous in plants, many of which have been used in traditional medicine for thousands of years. Of the many actions of coumarins, antioxidant, antiproliferative, and anti-HIV effects stand out. A large number of structurally novel coumarin derivatives have ultimately been reported to show substantial anti-HIV activity. Given that certain substituents are known to be required or increase their actions, the therapeutic potential of select coumarins is fairly obvious. There is considerable evidence that coumarins are important lead compounds for the development of antiviral and/or virucidal drugs against HIV. To describe the recent progress of the discovery, structure modification and structure-activity relationship of the potent anti-HIV coumarin derivatives is not an easy task. Nevertheless, it is useful to make some correlations of the available data which would help the researchers in discovering and developing of new active compounds used in drug design. The scientific society can modify the structures of many synthetic coumarin derivatives, which are lead compounds for anti-HIV agents, in order to design new anti-AIDS drugs. The molecular modeling methods may be a potential tool in the development of new anti-HIV agents. Molecular modeling systems provide powerful tools for building, visualizing, analyzing, and storing models of complex molecular systems (ie, inhibitor binding with receptor) that can help interpret structure-activity relationships.

## Figures and Tables

**Table 1:** A review of EC_50_ values and TIs (defined as LD_50_/IC_50_) for some coumarins.

Compound	EC_50_	TI	Reference

Suksdorfin	1,3 *μ*M	> 40	[1]
3′,4′-di-O-(–)-camphanoyl-	4 × 10^−4^ *μ*M*	136,719	[8]
(+)-*cis*-khellactone			
4-methyl-DCK lactam	0,00024 *μ*M	119,333	[9]
5-methoxy-4-methyl DCK	7, 21 × 10^−6^ *μ*M	> 2, 08 × 10^7^	[10]
3-hydroxymethyl-4-methyl-DCK	0,004 *μ*M in H9 cells	—	[11]
	0,024 *μ*M in PBMC**		
3-methyl-, 4-methyl-, and 5-methyl-	5, 25 × 10^−5^ *μ*M	2, 15 × 10^6^	[12]
3′,4′-di-O-(S)-camphanoyl-			
(3′R, 4′R)-(+)-*cis*-khellactone			
3-hydroxymethyl DCK	1, 87 × 10^−4^ *μ*M	1, 89 × 10^5^	[13]
4-methyl-3′,4′-di-O-(–)-	0,00718 *μ*M	> 21000	[1]
camphanoyl-(+)-*cis*-khelthiolactone			
3-bromomethyl-4-methyl-DCK	0,00011 *μ*M	189 600	[11]

*here IC_50_ but not EC_50_ is reported

**peripheral blood mononuclear cells
